# Kinetic Analyses of Data from a Human Serum Albumin Assay Using the ^li^SPR System

**DOI:** 10.3390/bios5010027

**Published:** 2015-01-19

**Authors:** Anja Henseleit, Carolin Pohl, Hans-Michael Kaltenbach, Karina Hettwer, Kirsten Simon, Steffen Uhlig, Natalie Haustein, Thomas Bley, Elke Boschke

**Affiliations:** 1 Institute of Food Technology and Bioprocess Engineering, Technische Universität Dresden, Dresden 01062, Germany; E-Mails: carolin.pohl@tu-dresden.de (C.P.); nataliehaustein@yahoo.de (N.H.); thomas.bley@tu-dresden.de (T.B.); elke.boschke@tu-dresden.de (E.B.); 2 QuoData GmbH, Prellerstraße 14, Dresden 01309, Germany; E-Mails: hans-michael.kaltenbach@quodata.de (H.-M.K.); hettwer@quodata.de (K.H.); uhlig@quodata.de (S.U.); 3 New diagnostics GmbH, Moosstraße 92c, Freising D-85356, Germany; E-Mail: simon@new-diagnostics.com

**Keywords:** surface plasmon resonance (SPR), human serum albumin (HSA), antibody, bivalent analyte

## Abstract

We used the interaction between human serum albumin (HSA) and a high-affinity antibody to evaluate binding affinity measurements by the bench-top ^li^SPR system (capitalis technology GmbH). HSA was immobilized directly onto a carboxylated sensor layer, and the mechanism of interaction between the antibody and HSA was investigated. The bivalence and heterogeneity of the antibody caused a complex binding mechanism. Three different interaction models (1:1 binding, heterogeneous analyte, bivalent analyte) were compared, and the bivalent analyte model best fit the curves obtained from the assay. This model describes the interaction of a bivalent analyte with one or two ligands (A + L ↔ LA + L ↔ LLA). The apparent binding affinity for this model measured 37 pM for the first reaction step, and 20 pM for the second step.

## 1. Introduction

Surface plasmon resonance (SPR) spectroscopy exploits the excitation of surface plasmons to follow molecular interactions in real-time without the need for additional molecular labels. A ‘surface plasmon’ represents a surface charge density wave at a metal-dielectric interface [1]. Light couples to the surface plasmon, and resonance occurs when its propagation constant equals the wave vector of light that is parallel to the interface [1,2,3]. The majority of SPR systems are based on the Kretschmann configuration [4,5], in which incident light passes through a prism that has a high refractive index, and is totally reflected at the metal-prism interface [2,3]. This generates a penetrating evanescent field which decays exponentially into the dielectric if the metal interface (typically gold) is sufficiently thin (less than 100 nm for visible and near infrared light) [2,3,6]. A binding-induced change in the dielectric refractive index leads to change in one or more light characteristics, such as the angle, phase, wavelength, or intensity. This latter change is needed to excite the surface plasmon wave [3]. Most SPR biosensors use the angle of the reflected light to monitor binding events. To achieve this, a wedge-shaped beam of monochromatic light is directed onto the metal interface to cover numerous angles of incidence [7]. The intensity of the reflected light reaches its minimum when the surface plasmon gets excited. The angle of incidence at which this dip occurs represents the sensor signal which is usually presented in a sensorgram as a function of time [3].

Human serum albumin (HSA) is exclusively synthesized in the liver, and is the most abundant plasma protein (60%, 40 mg/mL) [8,9,10]. The monomer is responsible for the preservation of pH and osmotic pressure, as well as for the transport of numerous substances such as metals, fatty acids, amino acids, hormones, vitamins and drugs [9,10,11,12]. HSA is a heart-shaped, 585 amino acids long protein (66.5 kDa) which consists of three homologue domains [12,13]. Due to its 17 disulfide bridges, this negatively-charged, single-chain protein has an average half-life of 19 days and remains stable at temperatures up to 60 °C as well as in pHs ranging from 7 to 9 [12,14].

About 20%–30% of the body’s hepatocytes are busy producing HSA at any given moment [15]. Therefore, HSA concentrations in plasma can be used as a reliable marker for the diagnosis and prognosis of various diseases [16]. For example, liver diseases are probable if the concentration of HSA in blood falls below the index value of 40 mg/mL [16,17]. HSA concentrations of approximately 20 mg/mL can indicate liver cirrhosis. Furthermore, HSA is one of the main nutrients for tumors. As a result, HSA levels in cancer patients may be low depending on the size and activity of their tumors [12]. Additionally, HSA can be used to test the viability of human hepatocytes cultivated* in vitro* [18,19,20]. Such cultivated liver tissues are of great interest in pharmacologic research because of their potential for predictive substance evaluation [20,21].

In this study, we used the ^li^SPR system to investigate the binding mechanism and determine the affinity of the HSA-antibody interaction involved. Affinity is normally represented as a dissociation constant, *K*_D_, which displays the concentration of an analyte at which half of the free ligand will be bound within a complex at equilibrium [22]. Thus, high *K*_D_ values signify low affinities. The affinities of antibody-antigen reactions typically appear in the micromolar to picomolar range [23].

The simplest model involves 1:1 binding. This model is based on the assumption that one analyte (A) molecule will bind to one ligand (L) molecule (A + L ↔ LA) [24]. However, we used polyclonal antibodies, which are a mixture of antibodies with different specificities and affinities for their antigen [25]. Thus, a more complex model is a better choice for imitating real binding. The heterogeneous analyte model, for example, assumes that two independent analytes will compete to bind to one ligand (A1 + L ↔ LA1, A2 + L ↔ LA2). Hence, two affinity constants will be determined. In contrast, the bivalent analyte model describes the interaction between a bivalent analyte to one or two ligands. In the first step of the binding reaction (A + L ↔ LA), the two ligand binding sites remain equivalent, and in the second step (LA + L ↔ LLA), cooperative effects contribute [24]. The second binding step depends on the flexibility of the monovalent bound ligand-analyte complex (LA) as well as its proximity to the next ligand molecule [26], and leads to stabilization of the resulting complex. The second association rate of the bivalent analyte model is reported in the unit Pixel^−1^s^−1^ instead of M^−1^s^−1^, because the local concentration of the analyte is taken into account.

In the proposed sensor, HSA was covalently attached to the carboxylated self-assembled monolayer (SAM) via its lysine residues by amine coupling procedure. The interactions of high-affinity antibodies with the immobilized HSA were investigated using the low-cost bench-top ^li^SPR system [7]. A lack of appropriate software for evaluating the computation of binding constants by the ^li^SPR system led to the implementation and evaluation of different algorithms for modeling the types of binding. To the best of our knowledge, this was the first time that kinetics studies of a HSA-antibody interaction were performed using SPR.

## 2. Experimental Section

### 2.1. Materials

HSA-specific antibodies were purchased from Biomol GmbH (Hamburg, Germany). HSA, 11-mercaptoundecanoic acid, and running buffer TBST (Tris buffered saline with Tween^®^ 20, pH 8.0) were purchased from Sigma-Aldrich Chemie GmbH (Steinheim, Germany). The amine coupling kit was purchased from GE Healthcare Europe GmbH (Munich, Germany). The reference ligand BSA (bovine serum albumin) was obtained from Merck KGaA (Darmstadt, Germany). All other chemicals were analytical grade and obtained from VWR International GmbH (Dresden, Germany).

### 2.2. SPR Measurements

The binding affinity and kinetic parameters of the HSA-antibody complex were investigated using the ^li^SPR system (capitalis technology GmbH, Berlin, Germany). The experiments were performed at 30 °C, with a flow rate of 5 µL/s. Levels of protein bound to the surface were measured in pixels, where 1 pixel roughly corresponded to 41 pg/mm^2^ [27].

Algorithms for fitting the binding models were implemented using the R software for statistical computing (www.r-project.org). The rate constants of the binding models were fitted locally and globally. In the case of local fitting, the rate constants were computed for each binding curve (antibody concentration). The global parameters applied to the whole data set (all binding curves).

#### 2.2.1. Preparation of the Gold Surface and SAM

The bare gold surfaces of the sensor chips (capitalis technology GmbH, Berlin, Germany) were first treated with UV/Ozone (UV/Ozone ProCleaner, NanoAndMore GmbH, Wetzlar, Germany) for 30 min and, afterwards, rinsed with pure ethanol.

The clean gold surfaces were immersed in 10 mM 11-mercaptoundecanoic acid overnight at 30 °C, after which they were thoroughly rinsed, sequentially, with ddH_2_O, ethanol, ddH_2_O, 100 mM HCl, 50 mM NaOH, 0.5% (v/v) SDS, and ddH_2_O, and then dried under a stream of nitrogen.

#### 2.2.2. Immobilization of HSA

For immobilization purpose, 10 mM sodium acetate (pH 4.5) was used as the running dielectric as well as for dilution of the molecules. The HSA molecules were immobilized covalently onto the sensor chip by the way of amine coupling of constituent lysine residues. To achieve this, solutions of 1.5 µM HSA were incubated for one hour in the presence of the previously-activated carboxylated surface. The remaining active groups on the carboxylated surface were then blocked with ethanolamine-HCl for 30 min. BSA was immobilized on the reference channel using the same strategy.

#### 2.2.3. Kinetic Measurements

The antibody samples were diluted in TBST to the desired concentrations and then injected onto the HSA-modified surface for 10 min. Dissociation was monitored by replacing the sample solution with TBST buffer for about 15 min. The degree of binding was determined by measuring the SPR signal at the end of the dissociation phase. Since this resulted in only incomplete dissociation, antibodies were finally removed from the HSA by injection of 100 mM glycine-HCl (pH 2.2) for 72 s.

## 3. Results and Discussion

**Figure 1:**
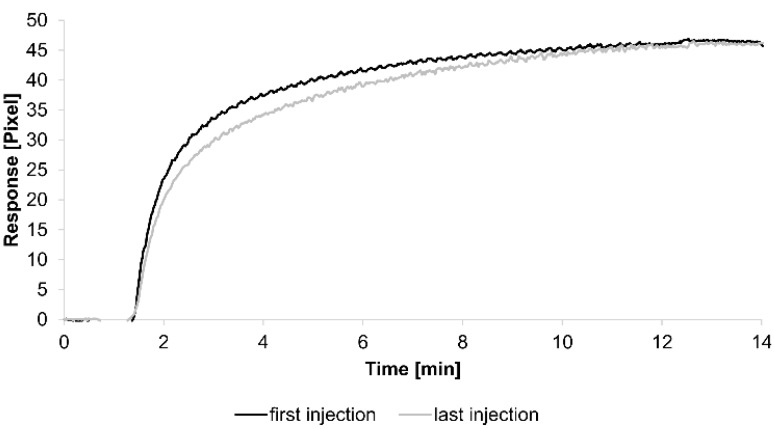
Sensorgrams showing the binding response of injections of 0.7 µM human serum albumin (HSA)-specific antibody before and after the kinetic measurements. Values resulting from the average of two sensing spots and the subtraction of reference surface signals from raw signal measurements are presented on the graph.

For the kinetic analyses, HSA was attached to a sensor chip by the amine coupling procedure. To show the reproducibility of our assay we injected 0.7 µM HSA-specific antibody immediately before and after the kinetic measurements (see below) (Figure 1).

The curves obtained from both injections were quite similar, demonstrating the reliability of the assay. Additionally, each tested concentration was analyzed using two sensing and two reference spots.

**Figure 2:**
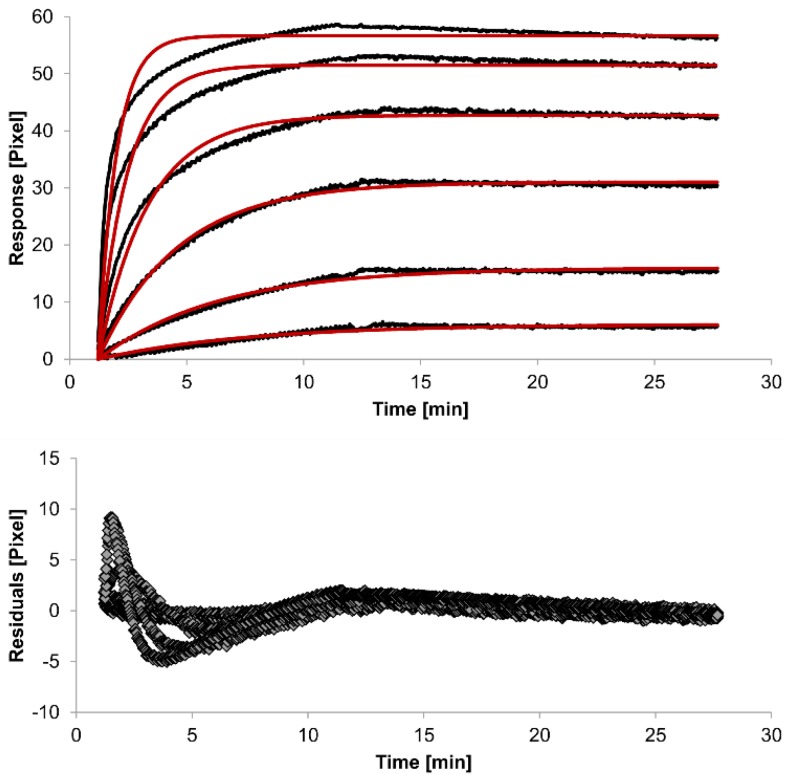
Kinetics of HSA-specific antibodies binding to immobilized HSA. Plots generated by applying the 1:1 binding model (red lines) overlay the plots associated with each antibody concentration (black lines; from top to bottom: 3.4 µM, 0.9 µM, 0.2 µM, 53.9 nM, 13.5 nM, 3.4 nM). Values resulting from the average of two sensing spots and the subtraction of reference surface signals from raw signal measurements are presented on the graph. The graph below depicts the residuals of the fits.

**Figure 3:**
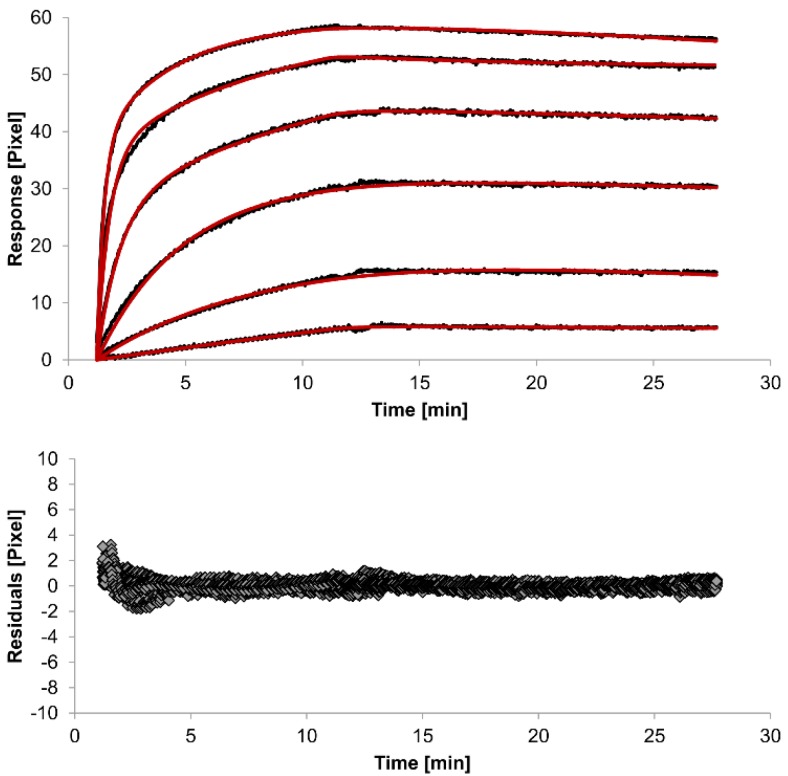
Kinetics of HSA-specific antibodies binding to immobilized HSA. Plots generated by applying the heterogeneous analyte model (red lines) overlay the plots associated with each antibody concentration (black lines; from top to bottom: 3.4 µM, 0.9 µM, 0.2 µM, 53.9 nM, 13.5 nM, 3.4 nM). Values resulting from the average of two sensing spots and the subtraction of reference surface signals from raw signal measurements are presented on the graph. The graph below depicts the residuals of the fits.

**Figure 4:**
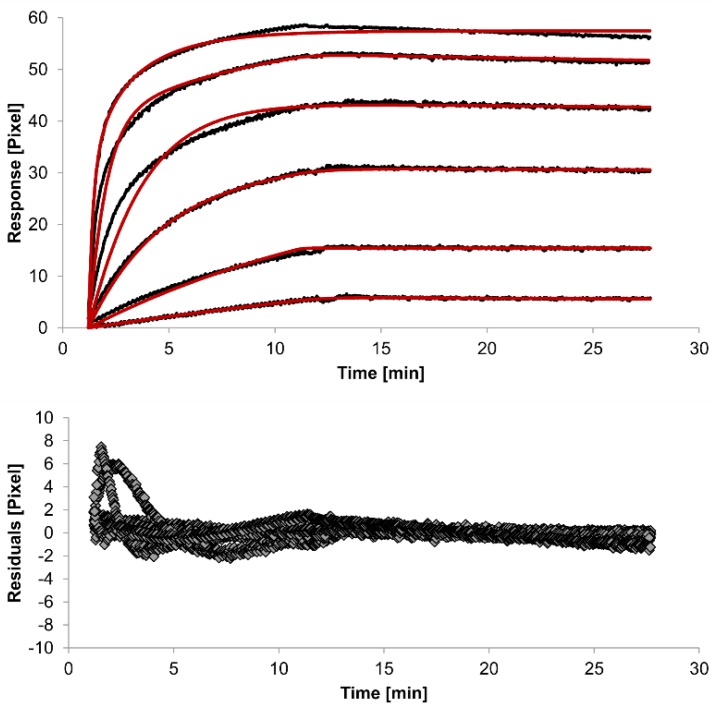
Kinetics of HSA-specific antibodies binding to immobilized HSA. Plots generated by applying the bivalent analyte model (red lines) overlay the plots associated with each antibody concentration (black lines; from top to bottom: 3.4 µM, 0.9 µM, 0.2 µM, 53.9 nM, 13.5 nM, 3.4 nM). Values resulting from the average of two sensing spots and the subtraction of reference surface signals from raw signal measurements are presented on the graph. The graph below depicts the residuals of the fits.

For kinetic measurements, antibody solutions ranging in concentration from 3.4 nM to 3.4 µM were sequentially injected. Data representing binding of the antibodies to HSA were evaluated using three different models. Figure 2, Figure 3 and Figure 4 show overlaid fits of different binding models, and the parameters are presented in Table 1.

A decision as to whether the fit is acceptable can be made based on visual inspection of the overlaid fit and the residuals. The residuals are the deviation between the experimental data and the modelled binding curve and provide an indication if the choice of the binding model is appropriate. The residuals should be roughly normally and independently distributed with zero mean.

The overlays and residuals of the 1:1 binding (Figure 2), heterogeneous analyte (Figure 3) and bivalent analyte (Figure 4) models, from local fitting of rate constants, show different results for goodness of fit. The main deviation between the models and the experimental data appeared during the association phase. Visual inspections of the results indicate that the experimental data were best fitted to the heterogeneous analyte binding model. Good results were also obtained with the bivalent analyte model whereas, the 1:1 binding model produced the worst fit.

Another way to measure goodness of fit is the Chi-squared (χ^2^) value, which reflects the differences between measured and expected (modelled) values (the smaller the value, the better the fit). Since the χ^2^ value depends on signal intensity, the values for the different immobilization levels should not be compared to each other. Nevertheless, the values of the χ^2^ are shown in Table 1.

**Table 1:** Kinetic parameters obtained from local fitting of the interaction between the immobilized HSA and HSA-specific antibody for each mathematical model. The average rate constant and standard error of the local fit values for each model are reported.

Interaction Model	*k*_a1_ [M^−1^s^−1^]	*k*_d1_ [s^−1^]	*k*_a2_ [M^−1^s^−1^]	*k*_d2_ [s^−1^]	χ^2^ [Pixel^2^]
1:1 binding	4 (± 1) ×10^4^	2.1 (± 0.2) ×10^−3^	/	/	1.8
heterogeneous analyte	2.3 (± 0.9) ×10^4^	1.2 (± 0.4) ×10^−2^	5 (± 2) ×10^4^	7 (± 4) ×10^−4^	0.1
bivalent analyte	3.1 (± 0.7) ×10^4^	1.8 (± 0.7) ×10^−4^	1.2 (± 0.7) ×10^6^ [Pixel^−1^s^−1^]	9 (± 5) ×10^−3^	0.8

The 1:1 binding model shows the highest χ^2^ value (1.8). This is not surprising because 1:1 binding is biologically implausible when an antibody is the analyte. In contrast, the heterogeneous analyte model best agrees with the experimental data. However, this model assumes that the analyte consists of two different HSA-specific species only, which might not be true for polyclonal antibodies. The χ^2^-value of the bivalent analyte model is 0.8, which lies between the values for the 1:1 binding and heterogeneous analyte models and represents a good data fit. Since antibodies are bivalent molecules, this model is most probable biologically. The resulting rate constants for the three models are summarized in Table 1. The average rate constant and the standard error with respect to the local fit (specific for every binding curve) are reported for each binding curve.

**Figure 5:**
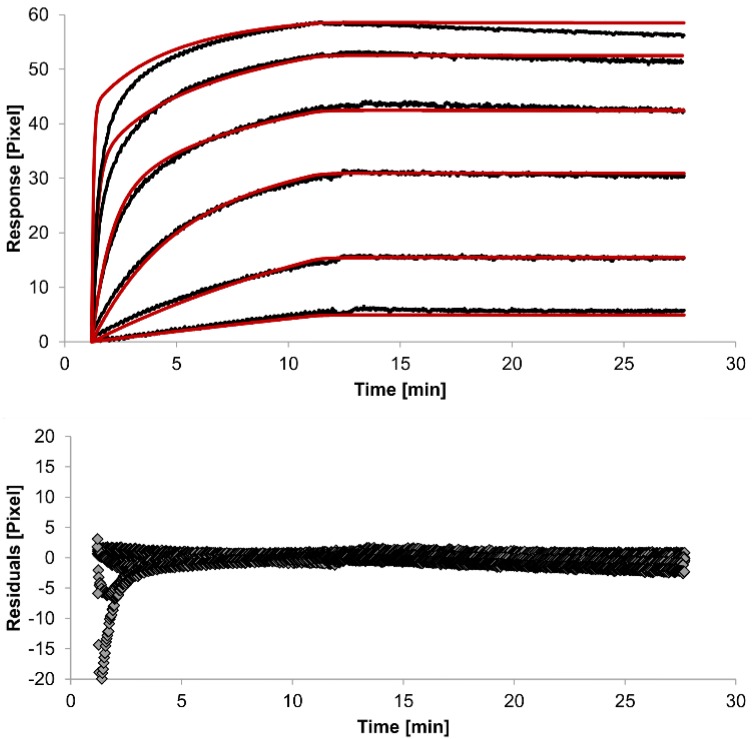
Kinetics of HSA-specific antibodies binding to immobilized HSA. Plots generated by globally applying the bivalent analyte model (red lines) overlay the plots associated with each antibody concentration (black lines; from top to bottom: 3.4 µM, 0.9 µM, 0.2 µM, 53.9 nM, 13.5 nM, 3.4 nM). Values resulting from the average of two sensing spots and the subtraction of reference surface signals from raw signal measurements are presented on the graph. The graph below depicts the residuals of the fits.

In spite of the above, the rate constants should be globally applied to all concentrations to achieve reliable results. Thus, global fitting of the rate constants was performed. Rate constants were calculated for the whole data set. Only the bivalent analyte model produced an acceptable global fit (Figure 5).

We observed an affinity (*K*_D_) of 37 pM (k_a1 _= 4.261 (±0.002) × 10^4^ M^−1^s^−1^, k_d1_ = 1.58 (±0.06) × 10^−6^ s^−1^) for the first (monovalent) binding step, with a χ^2^ value of 1.4 Pixels^2^. These values show that the global parameters fit the experimental data less accurately than the locally determined parameters. The affinity for the second (bivalent) binding step was measured at 20 pM (*k*_a2_ = 4.900 (±0.002) × 10^5^ RU^−1^s^−1^, *k*_d2_ = 8.420 (±0.007) × 10^−3^s^−1^). In fact, the dissociation rate for the bivalent binding step was much faster than for the monovalent binding step. This might be expected because monovalent binding occurs with free analytes whereas bivalent binding occurs with monovalently bound analytes [26]. The standard error of the globally determined parameters reflects the degree of uncertainty of the rate constants and are a measure for reliability. Considering all these factors, the bivalent analyte model seems to best represent the interaction between HSA and the antibody.

## 4. Conclusions

In this study, we performed kinetic measurements using our newly developed immunoassay which is based on surface plasmon resonance technology. Algorithms for calculating rate constants for sensorgram data from the ^li^SPR system were developed. Different binding models were evaluated, and the bivalent analyte model gave rise to the best fitting results. In the bivalent binding model, it is assumed that each antibody has two binding sites, one of which binds to the HSA molecule. The binding of the antibody’s second binding site to the HSA molecule stabilizes the complex.
